# Liposomal amphotericin B and renal safety: review of the evidence and clinical considerations

**DOI:** 10.1093/jac/dkaf473

**Published:** 2026-01-19

**Authors:** Johan Maertens, Rita Birne, Timothy Felton, Dionysios Neofytos, Martin Hoenigl

**Affiliations:** Department of Haematology, University Hospital Leuven, Leuven, Belgium; Department of Microbiology, Immunology and Transplantation, KU Leuven, Leuven, Belgium; Department of Nephrology, Unidade Local de Saúde de Lisboa Ocidental, Lisbon, Portugal; NOVA Medical School, Lisbon, Portugal; Division of Infection, Immunity to Infection and Respiratory Medicine, School of Biological Sciences, The University of Manchester, Manchester, UK; Transplant Infectious Diseases Unit, Division of Infectious Diseases, University Hospitals of Geneva, Geneva, Switzerland; Division of Infectious Diseases, Department of Medicine, Medical University of Graz, Graz, Austria; Translational Mycology Unit, Medical University of Graz, Graz, Austria

## Abstract

Invasive fungal infections continue to represent an important cause of morbidity and mortality in severely ill and immunocompromised patients. Liposomal amphotericin B (LAmB) has a significantly improved toxicity profile versus conventional amphotericin B deoxycholate and is recommended for a wide range of medically important opportunistic fungal pathogens. Although rates are significantly lower than with older formulations, nephrotoxicity with LAmB remains a concern. Risk factors for renal toxicity with LAmB include higher doses, longer duration of treatment, concomitant use of nephrotoxic agents and the presence of pre-existing kidney disease. Appropriate patient screening, individualized risk assessment, and patient monitoring may reduce the risk of renal toxicity. The prophylactic use of intravenous saline fluids is also recommended with LAmB to reduce the risk of nephrotoxicity. In addition, magnesium and potassium supplementation should be considered to reduce the risk of hypomagnesaemia and hypokalaemia, respectively. Alternate dosing strategies, including intermittent dosing and, for certain fungal infections, single-dose high-dose induction therapy, may be useful in minimizing nephrotoxicity, but additional research is necessary.

## Introduction

The introduction of amphotericin B deoxycholate (DAmB), a polyene with potent, broad-spectrum antifungal activity and low potential for drug–drug interactions (DDIs), into clinical practice in the late 1950s was a key moment in the management of life-threatening invasive fungal infections.^1,2^ Despite the favourable fungicidal properties of DAmB, its association with substantial adverse events (AEs), including potentially fatal cardiac or cardiorespiratory arrest, infusion-related reactions and significant nephrotoxicity, limit its clinical utility in adult patients to doses ≤1 mg/kg.^3^

**Figure 1.**
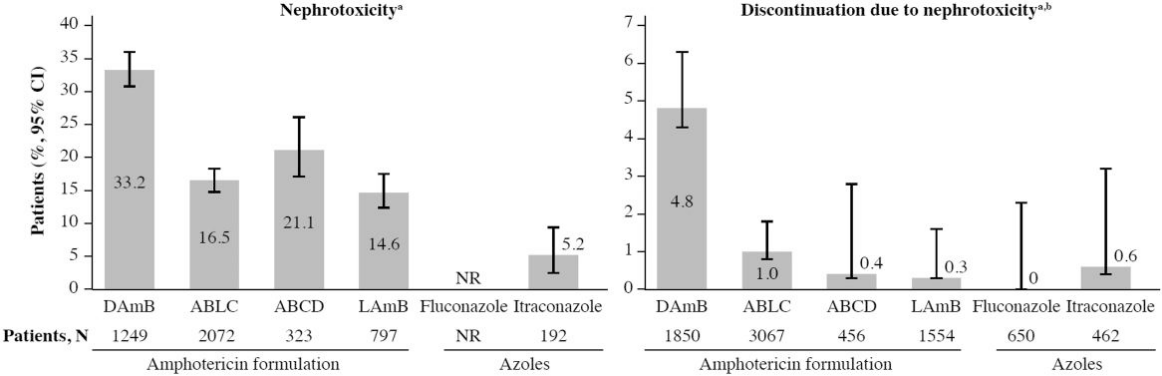
Rates of nephrotoxicity with lipid formulations of AmB during the treatment of invasive fungal diseases.^37^  ^a^Some references used other definitions of nephrotoxicity. ^b^All patients included in studies reporting any nephrotoxicity. NR, not reported.

Therapeutic options for the management of invasive fungal infections were limited to DAmB and the pyrimidine analogue 5-fluorocytosine until the 1990s, when additional agents were introduced into clinical practice. These included first-generation azoles, such as fluconazole and itraconazole, second-generation azoles (e.g. voriconazole, posaconazole, isavuconazole) and echinocandins (e.g. anidulafungin, caspofungin, micafungin), as well as lipid-based formulations of amphotericin B (AmB).^4^ However, these newer therapeutic options are not without limitations. Lipid-based formulations of AmB, such as liposomal AmB (AmBisome^®^; LAmB), AmB lipid complex (ABLC) and AmB colloidal dispersion (ABCD) have significantly fewer AEs than DAmB, including a lower incidence and later onset of nephrotoxicity (Table 1 and Figure 1).^5,37^ While the safety profile is improved, physicians may still be reluctant to prescribe these formulations to patients with pre-existing kidney disease due to their associated risk of acute kidney injury (AKI). The azoles are also associated with significant AEs, including hepatotoxicity and those related to frequent cytochrome P450-based DDIs, and often require therapeutic drug monitoring.^4,38^ While the echinocandins are considered to have the fewest AEs, their spectrum of activity is narrow compared with AmB and the azoles.^39^ Furthermore, unlike for the polyenes, the development of anti-fungal resistance is of growing concern for both the azoles and echinocandins.^40,41^

**Table 1.** Overview of renal toxicity in clinical trials involving LAmB for the prevention or treatment of invasive fungal diseases

Indication and reference	Compound	N	Median daily dose (mg/kg/d)	Mean duration of therapy (d)	Renal toxicity or glomerulotoxicity^a^ (%)	Discontinuations due to IRRs or renal impairment (%)
Treatment of invasive fungal diseases						
Andrew 2018^6^	LAmB	39	3 (range, 1-10)	6	21.2	18.2 (83.3% due to renal impairment and 16.7% due to IRR)
	DAmB	76	1 (range, 0.5-5)	3	14.3	21.4 (16.7% due to renal impairment and 66.7% due to IRR)
Burnett 2021^7^	LAmB	42	4	26	48	16.7
Burza 2022^8^	LAmB LAmB + miltefosine	75 75	5 5 + miltefosine 50 mg twice a day	N/A N/A	6.7 13.3	N/A N/A
Cagatay 2008^9^	LAmB	20	3	N/A	0	N/A
	ABLC	4	5	N/A	0	N/A
Cannon 2001^10^	LAmB	21	4.8 (range, 3-12)	16	19	N/A
	ABLC	46	5.3 (range, 3-13)	15	4.4	N/A
De Souza 2016^11^	LAmB	19	N/A	9	5.3	N/A
	DAmB	36	N/A	9	27.8	N/A
	ABLC	19	N/A	9	21	N/A
Dutta 2012^12^	LAmB	11	5	N/A	43	N/A
	DAmB	65	1	N/A	55	N/A
	ABLC	96	3–5	N/A	37.3	N/A
Falci 2015^13^	LAmB	105	3.3	8	2.4	N/A
	DAmB	236	0.78	8	11.5	N/A
	ABLC	90	4.31	8	7.2	N/A
Groll 2010^14^	LAmB	20	3	9.1	5	10
	LAmB + CAS	17	3 + 70 mg on Day 1, 50 mg thereafter	14.2	0	0
	CAS		70 mg on Day 1, 50 mg thereafter	12.9	0	5.5
Hachem 2008^15^	LAmB	51	10	N/A	5.9	N/A
	ABLC	30	5	N/A	10	N/A
Jarvis 2022^16^	LAmB + 5FC + FLC	420	10 mg/kg single dose	1	5.2	N/A
	DAmB + 5FC + FLC	422	1	7	5.9	N/A
Kuse 2007^17^	LAmB	202	3	15	3	N/A
	MICA	190	100 mg/kg	15	3	N/A
Leenders 1998^18^	LAmB	32	5	14.5	1.4	6.2
	DAmB	34	1	16.5	86	52.9
Luke 1998^19^	LAmB	165	5	15	30	N/A
	DAmB	93	0.6–1	16	48.3	N/A
Manzoni 2012^20^	LAmB	71	3–5	14	2.8	N/A
Miller 2004^21^	LAmB	18	3.7	7	44.4	N/A
	DAmB	51	0.81	7	70.6	N/A
	ABLC	34	4.7	7	41.2	N/A
Moghnieh 2016^22^	LAmB	89	5	N/A	23.6	N/A
Pasqualotto 2023^23^	LAmB	38	10 mg/kg single dose	1	11.8	N/A
		37	10 mg/kg on Day 1; 5 mg/kg on Day 3	3	26.7	N/A
		38	3	14	29.7	N/A
Prentice 1997^24^	LAmB	118	1	N/A	10	7.6
	LAmB	118	3	N/A	12	5
	DAmB	102	1	N/A	24	34.3
Rinaldi 2023^25^	LAmB	40	5 single dose; 14 pts received 3 mg/kg/d	14 in pts who received >1 dose	5.7	N/A
Rocha 2015^26^	LAmB	42	2.6	N/A	16.7	N/A
	DAmB	120	0.9	N/A	31.7	N/A
Shigemi 2011^27^	LAmB	22	3.2	18.3	27.3	N/A
Wade 2013^28^	LAmB	105	3	8.1	10.6	N/A
	ABLC	222	3	8.4	22.6	N/A
Walsh 1999^29^	LAmB	343	3	10.8	19	14
	DAmB	344	0.6	10.3	34	19
Walsh 2002^30^	LAmB	422	3	7	9.7	N/A
	VRC	415	12 mg/kg on Day 1, 6 mg/kg thereafter	7	11.3	N/A
Walsh 2004^31^	LAmB	539	3	14	11.5	N/A
	CAS	556	70 mg on Day 1, 50 mg/d thereafter	14	2.6	N/A
Yoshida 2020^32^	LAmB	53	3	14.4	3.9	N/A
	ITC	50	400 mg/d induction then 200 mg/d	14.0	2.0	N/A
Prophylaxis						
Annino 2013^33^	LAmB	48	Single dose (15 mg/kg)	Single dose (second dose 15 days after persistent neutropenia)	0	11.3 (due to mild IRR)
Cornely 2017^34^	LAmB	240	5 mg/kg twice weekly	22 days	6.3	N/A
El-Cheikh 2007^35^	LAmB	21	7.5 once weekly	N/A	24	9.5
Roman 2008^36^	LAmB	57	3	N/A	12	0

Adapted with permission from Tragiannidis A, et al. *Expert Opin Drug Saf* 2021; 20: 1061–74.^5^ Reprinted by permission of the publisher Informa UK Limited trading as Taylor & Francis Ltd, http://www.tandfonline.com.

5-FC, flucytosine; ABLC, amphotericin B lipid complex; CAS, caspofungin; DAmB, amphotericin B deoxycholate; FLC, fluconazole; IRR, infusion-related reaction; ITC, itraconazole; LAmB, liposomal amphotericin B; MICA, micafungin; N/A, not available; VRC, voriconazole.

^a^Renal toxicity is defined as any of the following: a doubling in the serum creatinine level from baseline; an increase of 88 mmol/L (1.0 mg/dL) in serum creatinine from baseline; or a > 50% decrease in the calculated creatinine clearance from baseline during study drug administration.

**Figure 2.**
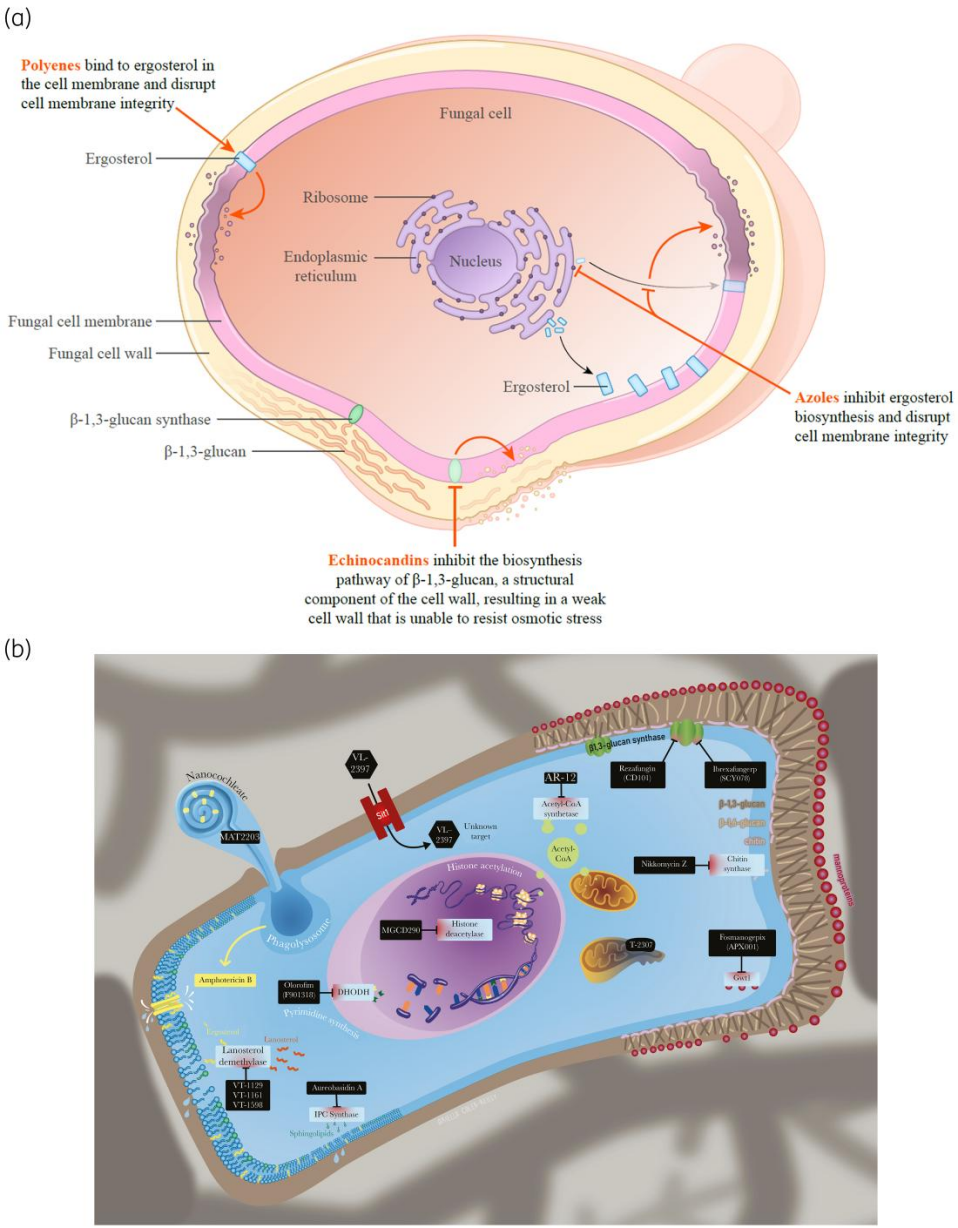
Mechanism of action of traditional and novel antifungal agents. (a) Traditional antifungal agents.^38^ (b) Developmental antifungal agents. Panel b reprinted from Rauseo AM, et al. *Open Forum Infect Dis.* 2020;7(2):ofaa016.^42^ Copyright ^©^ 2020 The Author(s). Published by Oxford University Press on behalf of Infectious Diseases Society of America.

Figure 2(a) summarizes the mechanisms of action of the currently available polyenes, azoles and echinocandins.^38^ While there are several new antifungals in various stages of clinical development with either novel mechanisms of action (e.g. manogepix/fosmanogepix and olorofim) or that demonstrate potential advantages over available antifungals (e.g. ibrexafungerp, rezafungin and the oral encochleated AmB formulation, MAT2203; Figure 2b),^39,42^ it will take years until they are broadly available for clinical use. Therefore, it is important to understand how to optimize the therapeutic use of currently available antifungal agents for the management of invasive fungal infections.

LAmB is currently recommended as a treatment option for invasive aspergillosis^43,44^ and candidiasis,^45^ is the drug of choice for the primary treatment of invasive mucormycosis (including cerebral disease)^44,46^ and is also an option for the empirical treatment of persistent febrile neutropenia.^44,47,48^ LAmB is also the recommended induction therapy for endemic mycoses including severe blastomycosis and disseminated histoplasmosis, as well as for cryptococcal meningitis and visceral leishmaniasis.^49–51^

The purpose of this review is to describe the renal safety of LAmB, to review the frequency of nephrotoxicity with LAmB and to evaluate strategies for optimizing patient and treatment selection to avoid renal issues in patients with or without pre-existing renal compromise.

### AmB mechanisms of action and nephrotoxicity

The underlying mechanisms of action and nephrotoxicity with AmB are closely related across all formulations. The mechanism of action of AmB involves the extraction of ergosterol from the membrane lipid bilayers of fungi and *Leishmania* protozoa, and the formation of membrane-permeabilizing pores that allow leakage of intracellular ions and organic substrates, which subsequently results in osmotic lysis as a result of perturbed membrane function.^2,5,52^

The therapeutic index of AmB is in part governed by its higher relative affinity for ergosterol in target pathogen membranes versus its affinity for the host membrane sterol, cholesterol, for which AmB has a 10-fold lower affinity.

AmB has been proposed to cause nephrotoxicity by several mechanisms.^2,5^ First, AmB may directly damage distal tubular membranes via its association with cholesterol and pore formation. Direct damage to these membranes may increase sodium influx in renal tubular cells and reduce the ability of tubular membranes to reabsorb electrolytes, resulting in hypokalaemia, hypomagnesaemia and renal tubular acidosis. Second, AmB may affect vasoconstriction of the afferent arteriole, either directly or indirectly, due to tubuloglomerular feedback, resulting in a subsequent reduction in renal perfusion and filtration. Finally, AmB may cause oxidative injury due to its impact on pro-inflammatory cytokines.^2,5^

### DAmB

With DAmB, these mechanisms have been shown to cause a transient and reversible decrease in glomerular filtration rate and an increase in serum creatinine (SCr) in up to 80% of patients.^53^ Clinically, AKI associated with DAmB typically presents as an increase in SCr 4–5 days after the initiation of therapy.^53^ More severe kidney injury can occur in the presence of diuretic-induced volume depletion or with the concomitant use of another nephrotoxic agent.^54,55^ Hypokalaemia and hypomagnesaemia usually develop 4–8 days following the initiation of therapy. However, these nephrotoxic events can also develop at any time during the course of therapy.^53,56^

Nephrotoxicity with DAmB is generally reversible, with renal function returning to normal following treatment discontinuation,^57,58^ although recurrent AKI can occur if treatment is reinstituted.^57^ Despite the general reversibility of DAmB-related nephrotoxicity, severe renal failure can occur, particularly in patients with pre-existing renal disease or those who concomitantly use other nephrotoxic or volume-depleting drugs. Furthermore, it is important to consider the total dose received, as permanent damage due to AmB-induced kidney injury has been reported in some cases, especially when the cumulative dose exceeds 5 g.^59^ However, data on the incidence, impact and timing of permanent kidney damage in patients using AmB therapy are limited.

Strategies for managing patients who experience nephrotoxicity with DAmB include dose reduction, treatment discontinuation, intermittent or continuous administration and ending concomitant use of other nephrotoxic agents; however, all of these strategies can compromise effective treatment.^5^

Sodium loading with intravenous administration of normal saline prior to initiation of DAmB is another recommended strategy for decreasing treatment-related nephrotoxicity. Dehydration and sodium depletion can impair glomerular filtration by interfering with the tubuloglomerular feedback mechanisms involved in constriction of the afferent arteriole.^5^ Consequently, saline loading, when tolerated, can reduce potential drug-induced renal toxicity. The quantity and duration of saline administration depend on the status of the patient.^60^ If patients are not dehydrated or volume depleted, 500 mL of saline can be administered immediately before DAmB. Alternatively, 250 mL of saline can be administered immediately prior to DAmB dosing and a further 250 mL administered after. If a patient is dehydrated, additional intravenous saline may be needed. A study of 77 consecutive patients who received DAmB reported that adequate hydration (1500 mL/m^2^ of body surface per day) and careful electrolyte supplementation helped minimize the risk of nephrotoxicity.^61^ In another study of 37 patients who received 50–100 mL of intravenous 10% NaCl during DAmB, none of the patients developed significant nephrotoxicity despite the concomitant use of other potentially nephrotoxic agents.^62^

Continuous infusion of DAmB may also be a useful strategy for minimizing nephrotoxicity because it avoids the high peak DAmB concentrations associated with conventional once-daily infusions that may cause AKI.^63–67^ While clinical studies have reported few differences in invasive fungal disease response between continuous and intermittent DAmB interventions,^63,66,67^ pharmacodynamic data from murine models indicate that peak serum DAmB levels may influence invasive fungal disease outcomes.^68^

### LAmB formulations

Several clinical trials, real-world studies, meta-analyses and systematic reviews have reported lower rates of nephrotoxicity with lipid formulations of AmB relative to DAmB (Table 1 and Figure 1).^5,13,28,37,69–72^ Data of meta-analyses and systematic reviews suggest that rates of nephrotoxicity with lipid formulations are approximately half those with DAmB,^5,37^ with rates ranging from 9% to 25% with LAmB and 12% to 50% with DAmB.^70^ LAmB has lower rates of severe toxicity than DAmB and ABLC, with an estimated incidence of severe nephrotoxicity of 11.5% with DAmB, 7.2% with ABLC and 2.4% with LAmB.^13^ Discontinuation rates due to nephrotoxicity appear to be much lower with LAmB, with rates of 4.8% among patients on DAmB, 1.0% among patients on ABLC and 0.3% among patients on LAmB.^37^

The functional sparing of the kidney with lipid formulations is thought to occur through several proposed mechanisms.^5^ First, lipid formulations are preferentially distributed into organs of the mononuclear phagocytic system (MPS). Lipid formulations also show enhanced stability and increased residence time of liposomally bound AmB in the bloodstream, with slow release of AmB into the circulation reducing the levels of circulating AmB available to bind host cell membranes. They are also thought to selectively transfer AmB from the lipid carrier to the fungal cell membrane and may result in decreased release of pro-inflammatory cytokines.

**Figure 3.**
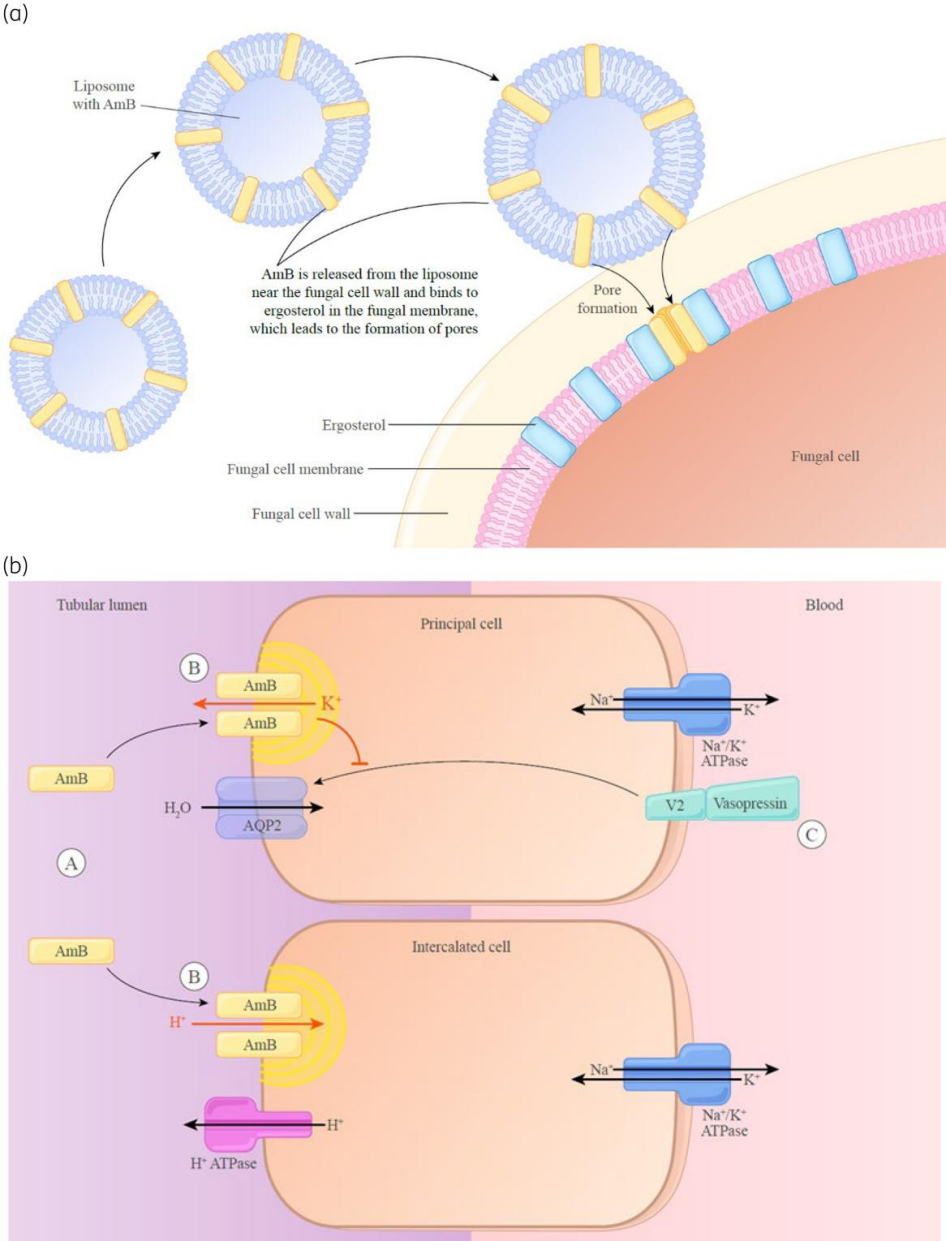
Mechanistic elements of LAmB. (a) Liposomal release of AmB at the fungal cell wall.^74^ (b) AmB and its off-target toxicities.^73^ A. AmB inserts into the membranes of principal and intercalated cells leading to the formation of pores. B. Pore formation disrupts the normal balance of ions by allowing the efflux of K^+^ from principal cells and back diffusion of H^+^ into intercalated cells. C. AmB also inhibits insertion of the aquaporin 2 (AQP2) channel induced by vasopressin.

The components of the liposome formulation associated with the improved renal safety profile of LAmB relative to DAmB include hydrogenated soy phosphatidylcholine, which ensures liposome stability and less release of free AmB into the circulation; distearoyl phosphatidylglycerol, which allows formation of an ion pair with the positively charged amino group of AmB; and cholesterol, which binds AmB within the liposome and helps prevent release of free AmB and its subsequent off-target toxicities (Figure 3).^2,73^

Given that formulations and manufacturing processes vary across lipid formulations of AmB, it is unsurprising that variable pharmacokinetic profiles and rates of renal AEs have been observed with these formulations (Table 1 and Figure 1).^5,13,28,37,69–72^ Data suggest that the small unilamellar liposomal preparation in LAmB has a prolonged residence time in plasma, has a high *C*_max_ and AUC and is absorbed slowly by the MPS, while ABLC formulations are efficiently opsonized by plasma proteins and more rapidly absorbed by the MPS, resulting in lower peak and AUC values.^5,75,76^ Several studies have demonstrated that LAmB formulations cause significantly less nephrotoxicity than ABLC at similar doses.^13,15,28,37,77^

## LAmB dosing and pharmacokinetics

LAmB dosing and pharmacokinetics are well-reported elsewhere and briefly summarized here.^1–3,78^

### LAmB dosing

The initial daily dose of LAmB for the treatment of life-threatening fungal diseases usually ranges between 3 mg/kg and 5 mg/kg of body weight, with a usual treatment duration of approximately 12.5–13 days.^31^ When LAmB is used for the empirical treatment of febrile neutropenia, the recommended dose is 3–5 mg/kg/d of body weight.^78^ In contrast, the recommended starting dose of LAmB for the treatment of mucormycosis is 5–10 mg/kg/d.^46^ The slow escalation of dosing should be avoided. The duration of LAmB treatment for mucormycosis should be individualized but is typically 6–8 weeks, although longer durations may be required for deep-seated infections or cases that occur during prolonged courses of chemotherapy or neutropenia. A total dose of 21–30 mg/kg of body weight given over 10–21 days is recommended for the treatment of visceral leishmaniasis.^78^

Typically, the infusion duration for doses up to 5 mg/kg/d should be approximately 30–60 min. When doses greater than 5 mg/kg/d are required, it is recommended that the infusion duration be increased to 2 h.^78^ Furthermore, a single dose of 10 mg/kg and daily doses up to a maximum of 10 mg/kg/d have been used in clinical trials and are recommended by guidelines for clinical practice. However, while multiple trials have confirmed greater safety and tolerability of the single high-dose regimen over prolonged administration of the standard daily dose (3.0–5.0 mg/kg), trials evaluating the safety of prolonged daily treatment with doses greater than 5 mg/kg are limited.^2,16,23,46,79–81^ Individualized patient assessments of the potential benefits of higher doses versus the increased risk of AEs with higher doses, such as nephrotoxicity, are therefore required.^78^

#### LAmB dosing for patients with renal impairment

LAmB has been administered to patients with pre-existing renal impairment at starting doses ranging from 1 to 3 mg/kg/d in clinical trials without the need for adjustments to dose level or frequency.^78,82–85^ Dose adjustments for patients undergoing haemodialysis or filtration procedures are not required.^86,87^ Briefly, additional factors that could influence dose selection include patient body weight and concomitant extracorporeal membrane oxygenation.^74,88–91^

### LAmB pharmacokinetics

Preclinical data show that LAmB, in comparison to DAmB, when given at higher doses, resulted in enhanced plasma exposure and increased drug disposition in the lungs and the CNS, equal or improved antifungal efficacy and reduced nephrotoxicity, without relevant new toxicities.^1^

The ability to achieve higher exposure levels with LAmB was confirmed in dose-finding studies in adult patients with febrile neutropenia (1.0, 2.5, 5.0 or 7.5 mg/kg LAmB infused over 1 h)^92^ and in patients with neutropenia infected with *Aspergillus* spp. or other filamentous fungi (7.5, 10.0, 12.5 or 15.0 mg/kg/d of AmB as LAmB administered as a 2-h infusion).^79^ Both studies demonstrated a non-linear dose relationship, consistent with saturation of the reticuloendothelial uptake as the major clearance pathway, particularly at doses exceeding 7.5 mg/kg/d.^79,92^ The greatest values for LAmB *C*_max_ and AUC occurred following the administration of 10 mg/kg/d and declined at 12.5 and 15 mg/kg/d, suggesting a possible change in the elimination mechanism with doses exceeding 10 mg/kg/d.^79^ Drug accumulation was not observed.^79,92^

It is possible that LAmB clearance mechanisms relate to enhanced uptake by the reticuloendothelial system, potentially via low-affinity lipid receptors that clear liposomes at relatively high plasma concentrations. Such enhanced uptake may explain the higher concentrations of AmB in the liver, spleen and bone marrow and may have important therapeutic implications for the treatment of fungal infections in tissues outside the reticuloendothelial system. For example, data suggest that the upper dose limit for patients with infections in the CNS is 10 mg/kg because higher LAmB doses are not accompanied by corresponding increases in LAmB exposure. One example may be *Candida* meningoencephalitis, an infection in which LAmB exposure is the principal determinant of its efficacy and its penetration into the CNS is determined by its *C*_max_.^79^

Mass-balance studies of LAmB (2 mg/kg) and DAmB (0.6 mg/kg) in healthy volunteers showed triphasic plasma profiles for both formulations.^93,94^ Relative to DAmB, LAmB had a disproportionately higher *C*_max_ (mean *C*_max_, 22.9 versus 1.4 μg/mL, respectively) and smaller volume of distribution at steady state. The renal and faecal clearance rates of LAmB were 10-fold lower than with DAmB, while overall clearance rates were comparable. In total, <10% of the LAmB dose was excreted unchanged, and no metabolites were identified. Dosing with LAmB was associated with lower exposure to both unbound and non-liposomal AmB, with the majority of AmB remaining liposome associated (97% at 4 h, 55% at 168 h).^93^

## Renal safety of LAmB

To date, no studies have formally investigated the relationship between LAmB exposure and renal toxicity.^3^ However, studies comparing LAmB 3 mg/kg/d with higher doses (5, 6, 10 or 15 mg/kg/d) have demonstrated that higher doses are associated with higher incidence rates of increased SCr, hypokalaemia and hypomagnesaemia.^78,95^ Historically, investigating the association between LAmB exposure and renal toxicity has been challenging due to the lack of a standardized method for quantifying AmB levels, but recent evidence suggests that an analytical method using an ultra-performance liquid chromatography-photodiode array provides reliable estimates of AmB in plasma over a wide range of concentrations,^96^ providing opportunities for additional research.

### Results of clinical trials

#### Dose-finding studies

The renal tolerability of LAmB has been documented in a dose-ranging study, which evaluated doses of 1.0–7.5 mg/kg/d in patients with neutropenia, and a maximally tolerated dose (MTD) study, which evaluated doses of 7.5–15 mg/kg/d in patients with infections due to *Aspergillus* or other filamentous fungi.^79,92^ In the dose-ranging study, in which patients received LAmB 1.0–7.5 mg/kg/d for a duration of 8–11 days, there were no significant changes in SCr, potassium and magnesium levels in any dosing cohort, and no overall increase in SCr levels was noted.^79,92^ Only 3 (8%) of the 36 patients included in the study experienced a greater than 2-fold increase in SCr level, even though many were receiving concomitant potentially nephrotoxic agents, including gentamicin, vancomycin, foscarnet and cyclosporin.^92^ The MTD study demonstrated that the MTD of LAmB was at least 15 mg/kg/d.^79,92^ In this study, the number of LAmB infusions ranged from 1 to 83, with a median duration of therapy ranging from 9 days in the 15 mg/kg/d group to 24 days in the 7.5 mg/kg/d group. A total of 9 (20%) of 44 patients discontinued the drug due to AEs. Fourteen patients (32%) had an SCr at least two times greater than their SCr at baseline, although no significant dose-dependent trends were noted regarding changes in SCr. However, there were more cases of severe hypokalaemia (≤2.5 mEq/L) in patients receiving >10 mg/kg/d than in those receiving ≤10 mg/kg/d (35% versus 0%; *P* = 0.006). There were also eight cases of hypomagnesaemia reported in the study, but no dose-related association was reported.

Overall, serial assessments of SCr concentrations showed relatively little glomerular nephrotoxicity at dosages as high as 7.5–15.0 mg/kg/d.^79,92^ Although there was little evidence of dose-dependent glomerular dysfunction in this study of high-dose LAmB, patients receiving higher dosages of LAmB showed a significantly higher rate of hypokalaemia. These results suggest that the renal tubular epithelium may be more sensitive to high LAmB dosages than cells mediating glomerular filtration, including podocytes and the endothelial cells of the glomerular capillaries.

#### AmBiLoad

In the AmBiLoad trial, a study of patients with invasive mould infection randomized to receive LAmB at either 3 or 10 mg/kg/d for 14 days followed by 3 mg/kg/d, more patients on 10 mg/kg/d experienced an increase in SCr to twice the baseline value than patients on 3 mg/kg/d (31% versus 14%; *P* < 0.01).^81^ Grade 3 hypokalaemia, defined as a blood potassium level <3.0 mmol/L, also occurred more frequently in the high-dose group (30% versus 16%; *P* = 0.015), although no significant between-group differences in the rate of grade 4 hypokalaemia (potassium <2.5 mmol/L) were observed.

More patients on the higher dose of LAmB discontinued the study drug due to AEs compared with the lower dose (32% versus 20%; *P* = 0.035), with increases in SCr, abnormal liver function tests and hypokalaemia as the AEs most commonly prompting drug discontinuation in both groups. Based on the results of this study, international guidelines adopted 3 mg/kg/d as the recommended dose for the treatment of *Aspergillus* infection.^48,97^

#### AmBiDex

The safety of LAmB was evaluated in the AmBiDex study, a multicentre trial evaluating weekly high-dose LAmB use in 21 non-immunocompromised critically ill patients with sepsis with ICU-acquired *Candida* colonization at multiple sites.^98^ All patients in the study received weekly high-dose LAmB (10 mg/kg/week) for 2 weeks. Eighty percent of patients also received concomitant nephrotoxic agents.

Few patients experienced electrolyte disturbances or severe renal toxicity. Although no patients experienced severe hypokalaemia (<2.5 mmol/L), 5 (23.8%) patients demonstrated an increase in SCr, with a 2-fold and 3-fold increase from baseline in 3 (14.3%) and 2 (9.5%) cases, respectively. No patients required renal replacement therapy. When patients receiving high-dose LAmB were compared with matched control participants, no significant increases in SCr levels were noted.

#### AMBITION

The AMBITION study evaluated the safety of a single high dose of LAmB for the treatment of cryptococcal meningitis in HIV-positive patients in five African countries.^16^ In this study, 844 patients were randomized to either a single high dose of LAmB (10 mg/kg) on Day 1 plus flucytosine (100 mg/kg/d) and fluconazole (1200 mg/d) for 14 days or the current treatment regimen recommended by the WHO (control; DAmB 1 mg/kg/d plus flucytosine 100 mg/kg/d for 7 days followed by fluconazole 1200 mg/d for 7 days).

At 10 weeks, LAmB was noninferior to the WHO-recommended treatment regimen. Fewer instances of grade 3 or 4 anaemia (13.3% versus 39.1%; *P* < 0.001), hypokalaemia (1.4% versus 6.4%; *P* < 0.001) and overall AEs (50.0% versus 62.3%; *P* < 0.001) were observed in the LAmB group compared with the control group. A lower mean relative increase in SCr from baseline to Day 7 (20.2% LAmB versus 49.7% control; *P* < 0.001) was also observed.^16^ Given the efficacy and tolerability of LAmB in this trial, this is now the recommended treatment for cryptococcal meningitis by the WHO.^99^

#### AmBizygo

AmBizygo was a prospective pilot study of the effects of 1 month of treatment with high-dose (10 mg/kg/d) LAmB for the initial treatment of mucormycosis (*N* = 40).^80^ The median cumulative LAmB dose was 161 mg/kg, with a median dose per day of 9.5 mg/kg/d. LAmB was administered at a 10 mg/kg/d dose for a median of 13.5 days.

A total of 16 out of 40 (40%) patients experienced SCr doubling, and 10 (63%) recovered normal kidney function, 4 (25%) died and 2 (12%) did not recover within 3 months of end of treatment. Renal toxicity was more frequent among patients with diabetes, possibly due to the increased risk associated with diabetic nephropathy. Hypokalaemia (potassium <3 mmol/L) was also experienced by 16 (40%) patients in the study. Based on this and the results of other studies, guidelines have adopted 5–10 mg/kg/d as the recommended dose range for treatment of mucormycosis and other rare mould infections.^46,100^

#### Empirical therapy in persistent fever and neutropenia

Several randomized, multicentre studies compared empirical therapy with LAmB and DAmB in adult or paediatric patients with fever and neutropenia who were not responding to treatment with broad-spectrum antibiotics.^24,29,77^ A combined analysis of two randomized, open-label, multicentre evaluations reported greater rates of clinical success (defined as a minimum of 3 days with fever <38°C, continuing in the study until its end and recovery of neutrophils to 0.5 × 10^9^/L) with LAmB (58% and 64% for 1.0 and 3.0 mg/kg/d, respectively) compared with DAmB (49% for 1.0 mg/kg/d) treatment. Importantly, fewer LAmB-treated patients not receiving concomitant nephrotoxic drugs experienced SCr doubling (0% and 3%, respectively) compared with DAmB-treated patients (23%) (*P* < 0.01).^24^ Additional randomized, double-blind trials have reported similar efficacy and safety observations.^29,77^ Furthermore, subsequent large-scale clinical trials comparing LAmB with new antifungal compounds (e.g. voriconazole and caspofungin) have confirmed the efficacy, tolerability and clinical utility of LAmB in this setting.^30,31^

#### Antifungal prophylaxis in AML

The safety of a single very high dose of LAmB (15 mg/kg, 6-h infusion) for antifungal prophylaxis was evaluated in an open-label study of 48 patients with AML.^33^ All patients received LAmB 15 mg/kg within 24 h after the cessation of chemotherapy; a second 15 mg/kg dose was administered to patients with persistent profound neutropenia (<100 neutrophils/μL) 15 days after the first dose. While prolonged serum and tissue LAmB levels were observed, only four patients (7.5%) experienced grade 1 or 2 increases in SCr, with no cases of grade 3 or 4 increases reported. Grade 3 hypokalaemia was reported in six patients (11%), within 10 days of the first LAmB infusion (*n* = 5) or within 5 days of the second infusion (*n* = 1), all of which were corrected with potassium supplementation.

#### Treatment of endemic mycoses

LAmB is the recommended induction therapy for several common endemic mycoses including blastomycosis and histoplasmosis, though clinical trials evaluating LAmB in endemic mycoses are limited.^51^ One seminal double-blind, multicentre, randomized clinical trial compared the safety and efficacy of LAmB (3.0 mg/kg/d) versus DAmB (0.7 mg/kg/d) for 14 days in 81 patients with AIDS and disseminated histoplasmosis.^101^ Clinical success was achieved in a greater percentage of LAmB-treated patients (88%) compared with DAmB-treated patients (64%; *P* = 0.014). Patients who received LAmB also demonstrated lower rates of nephrotoxicity, defined as SCr levels greater than twice the baseline level, compared with those who received DAmB (9% versus 37%, respectively; *P* = 0.003). These results informed the current treatment regimen recommended by the WHO: LAmB at 3 mg/kg/d for 14 days.^101,102^

To further optimize safety, an additional trial compared the efficacy and safety of 3 LAmB treatment regimens in 118 patients with AIDS and disseminated histoplasmosis: a single high dose of 10 mg/kg on Day 1; one dose (10 mg/kg) on Day 1 followed by one dose (5 mg/kg) on Day 3; or 3 mg/kg/d for 14 days (control).^23^ The single-dose treatment arm yielded a greater clinical response (84%) than the two-dose and control arms (69% and 74%, respectively). Importantly, though not statistically significant, lower rates of kidney toxicity in accordance with the Kidney Disease: Improving Global Outcomes (KDIGO) classification were also observed on Day 14 in the single-dose group (11.8%) compared with the two-dose and control groups (26.7% and 29.7%, respectively).

### Clinical considerations

While nephrotoxicity is the primary treatment-limiting AE associated with LAmB, the risk of adverse renal effects varies depending on the dose and duration of therapy and a wide range of patient-specific factors. Nonetheless, when renal toxicity does occur with LAmB, most cases are generally manageable and reversible. Of note, a recent study in the real-life setting found a low incidence of renal-related adverse drug reactions in LAmB-treated patients, suggesting manageability of LAmB’s renal effects.^103^

As indicated in Table 1 and Figure 1, rates of nephrotoxicity reported in clinical trials of LAmB are highly variable and range from 1.4% to 44.1%.^5,37^ These differences could be attributable to variability in study populations, lack of randomized data, and exposure to confounders, such as concomitant nephrotoxins. That the highest nephrotoxicity rate of 44.1% was observed in a cohort of haematopoietic stem cell transplant recipients outlines the potential role of concomitant nephrotoxic agents.^21^ Clinical studies also have used varying definitions of nephrotoxicity. Some investigators may define nephrotoxicity based on absolute SCr levels, while others may define nephrotoxicity based on a relative change from baseline.^28^

The use of different classification systems for nephrotoxicity may also result in different rates of nephrotoxicity. The Risk, Injury, Failure, Loss of kidney function, and End-stage kidney disease and the Acute Kidney Injury Network systems are validated for determining the incidence of acute kidney failure and enabling prognostic stratification. Studies have shown variability in their reporting of concurrent medical conditions and the use of concomitant medications, which can have a significant impact on the development of nephrotoxicity. Given the impact of acute illnesses on kidney injury and changes in glomerular filtration, differences in the type and severity of the underlying acute illness may also affect rates of nephrotoxicity. For example, fungal angio-invasion may lead to micro-abscesses in the kidney, resulting in renal infarcts and, ultimately, renal dysfunction.^104^ Certain fungal metabolites, such as ochratoxins, which are byproducts of infections of certain *Aspergillus* species, have also been recognized to be nephrotoxic.^105^

#### Impact of nephrotoxicity on LAmB treatment discontinuation

The variable rates and severities of LAmB-associated nephrotoxicity, which arise from differences in patient populations, disease states, LAmB dosing and duration and concomitant medications, can lead to variations in therapy outcomes. This may include differences in the proportion of patients who complete therapy as prescribed or switch to a different therapy.

A 10-year cohort study of adult patients who underwent allogeneic haematopoietic cell transplants with proven or probable invasive mould infections (IMI) evaluated the duration of, and changes in, antifungal therapy.^87^ Sixty-one patients with 66 cases of IMI were identified. Overall, patients continued treatment for 157 days, and those patients who were alive on Day 84 after their IMI diagnosis continued therapy for 213 days. LAmB was used by approximately 37% of the study population. The most frequently used first-line treatment was a mould-active azole (48.7%), followed by LAmB (37.2%) and echinocandins (14.1%).

Changes in antifungal treatment occurred in 57 patients (86.4%). More than one reason could have been given for each treatment change. Concerns about toxicity were most common among patients using azoles. Among the 85 total reasons cited for treatment changes by patients using azoles, toxicity issues were cited 43 times, 23 of which represented concerns about hepatotoxicity. Among the 53 reasons cited for changes to therapy among patients using LAmB, the most frequent were clinical efficacy (60.4%), including lack of clinical improvement, a change to a targeted treatment or clinical suspicion of invasive aspergillosis; toxicity (20.7%); logistical reasons (8.6%); and no reason recorded (8.6%). Toxicity issues were cited as the reason for changes in therapy 12 times, 10 of which were concerns related to nephrotoxicity. All-cause mortality 1 year after IMI diagnosis was four times higher among patients whose treatment was changed more than twice during the first 6 weeks of therapy, likely because patients who required more treatment changes may have been sicker and had more comorbidities, although a potential negative effect of treatment changes and/or toxicities cannot be excluded. Additional research into the impact of nephrotoxicity with LAmB on treatment changes and outcomes is needed.

## Risk management strategies

### Risk assessment

**Figure 4.**
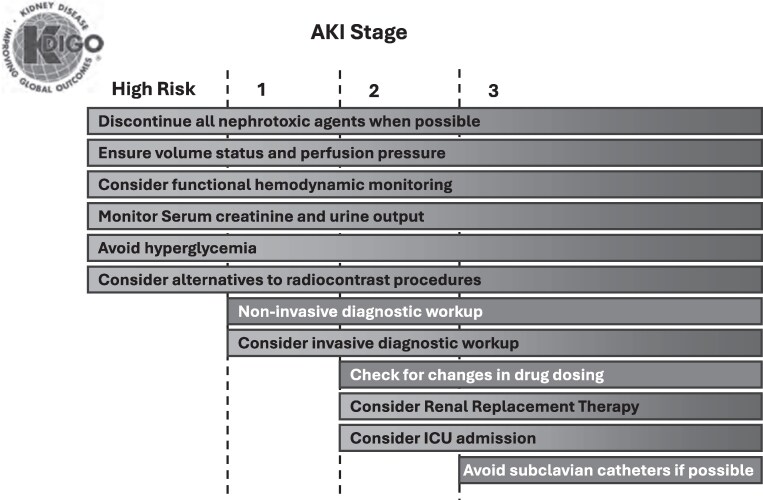
Stage-based management of AKI. Shading of boxes indicates priority of action—solid shading indicates actions that are equally appropriate at all stages whereas graded shading indicates increasing priority as intensity increases. ICU, intensive care unit. Reprinted from Kidney Disease: Improving Global Outcomes (KDIGO) Acute Kidney Injury Work Group. *Kidney Int Suppl* 2012; 2(Suppl): 1–138.^107^ Copyright ^©^ 2012 International Society of Nephrology. Published by Elsevier Inc.

Patients at risk for AKI include those exposed to factors that damage the kidneys, such as sepsis and the use of nephrotoxic drugs, or those who have characteristics that increase their susceptibility to AKI, such as dehydration, advanced age, diabetes or cancer.^106^ SCr and urine output should be assessed to evaluate patients at increased risk for AKI, and the frequency and duration of screening should be individualized based on their baseline risk (Figure 4).

According to the KDIGO 2012 guidelines for managing AKI, volume status and perfusion pressure should be ensured, the use of nephrotoxic agents should be discontinued when possible, hyperglycaemia should be avoided and alternatives to radiocontrast procedures should be considered.^106^

Current evidence suggests that risk factors for LAmB-induced nephrotoxicity include male sex, increased weight, higher LAmB doses, the presence of pre-existing kidney disease and the concomitant use and duration of nephrotoxic therapies.^95,108,109^ Note that the presence of AKI is not a contraindication to initiating LAmB therapy in patients who have a clinical diagnosis of a potentially life-threatening invasive fungal infection.^85^

### Reducing the risk of and managing renal AEs during LAmB therapy

According to LAmB labelling, regular laboratory evaluation of serum electrolytes, including potassium and magnesium, and monitoring of renal, hepatic and haematopoietic function should be conducted, at least once weekly, particularly among patients receiving concomitant nephrotoxic medications.^78^ Renal function should be monitored daily, and appropriate supportive care should be provided to help optimize patients’ kidney function.^85^ If a clinically significant reduction in renal function or worsening of other laboratory parameters occurs, consider reducing the dose, interrupting treatment or discontinuing therapy. Volume depletion, if present, must be corrected prior to LAmB use. The kidney is particularly sensitive to the effects of hypoperfusion and to hypervolaemia leading to congestion pressure. Consequently, systemic haemodynamics should be optimized to maintain adequate renal perfusion and perfusion pressure. Patients with sepsis should be managed with early and appropriate antibiotic therapy, which also may prevent further kidney injury. Other nephrotoxins should be avoided.

Clinicians should discuss the risks and benefits of starting LAmB within the clinical team and with the patient and/or their caregivers.^85^ Additional discussions of the risks and benefits of LAmB should occur if AKI develops and when the causative agent of the infection is microbiologically confirmed.

Pre-treatment administration of isotonic fluids (e.g. 500 mL divided before and after LAmB administration) and magnesium and potassium supplementation is generally advised.^5,61^ Fluid infusions after the development of LAmB-induced nephrotoxicity may also be associated with recovery from AKI, particularly in patients with early-stage AKI. In one retrospective study, 91% of patients with stage 1 AKI who received >10 mL/kg/d of fluid for 7 consecutive days after AKI onset recovered from AKI, compared with a recovery rate of 50% among patients who received less fluid infusion.^108^ Regardless of AKI stage, daily fluid infusion volume positively correlated with the incidence of AKI recovery.

Patients prescribed LAmB frequently require potassium supplementation during treatment. Renal solute wasting is generally more evident in those with preserved glomerular filtration rate than in those with severe AKI or chronic kidney disease. Early intervention with potassium prophylaxis should be considered.^110^ Patients receiving high doses of LAmB (e.g. >10 mg/kg/d) may receive pre-emptive intensive potassium supplementation to prevent the development of hypokalaemia.^79^

Patients who develop distal renal tubular acidosis require alkali replacement, and magnesium supplementation is indicated if magnesium wasting is observed. Note that electrolyte abnormalities may continue for weeks after LAmB discontinuation.

### Alternate dosing strategies

Various dosing strategies have been developed to limit AmB-induced nephrotoxicity. One strategy is to give AmB as a continuous infusion rather than a 2- to 4-h infusion; however, while there is some suggestion that a continuous infusion may limit nephrotoxicity,^63–67^ enthusiasm for this strategy is tempered by the potential loss of antifungal activity.^68,111^ AmB exhibits concentration-dependent antifungal activity, and continuous infusion of low-doses of AmB could result in suboptimal protection for some patients with invasive fungal infections.

Another strategy is the administration of LAmB on alternate days, rather than daily, or even weekly dosing.^112–114^ This option is better tolerated than daily dosing and might reduce nephrotoxicity without sacrificing efficacy in stable patients. This approach may be particularly useful for primary antifungal prophylaxis, but LAmB is not approved for this indication.^1^ Additional data on its prophylactic use are also needed.

#### Inhaled LAmB delivery

Aerosolized delivery may be an attractive option for preventing fungal lung infections, promising minimal systemic exposure and high local drug exposure, which are useful because the effects of AmB in the lungs depend on its dose and concentration.^1^ Limited systemic exposure may also reduce the risk of nephrotoxicity. Although the use of aerosolized LAmB as treatment or prophylaxis has been reported in a number of studies,^115–117^ its clinical use is investigational and is not currently licensed, and additional data on its use are needed.

## Renal considerations with other antifungal therapies

### Pyrimidine analogues

There are no reports of intrinsic nephrotoxicity with flucytosine.^5^ As flucytosine is primarily eliminated through glomerular filtration, dose adjustment is required in patients with impaired renal function, and caution is required during coadministration with other drugs that impair glomerular filtration. The most significant AEs are myelosuppression and hepatic toxicity, including elevation of serum transaminases and alkaline phosphatase, occurring in up to 25% of patients.^5^ Given the impact of reduced renal function on elimination, observed myelosuppression and hepatic toxicity, therapeutic drug monitoring is required for flucytosine; close monitoring of haematological and hepatic parameters is also required. Flucytosine is contraindicated in patients with known complete dihydropyrimidine dehydrogenase deficiency.

### Triazoles

Although triazoles are not generally considered to have direct nephrotoxic effects,^5^ intravenous cyclodextrin formulations of several triazoles are eliminated by glomerular filtration. A significant challenge with triazole-based therapy is its propensity to result in DDIs; these interactions can indirectly contribute to nephrotoxicity and renal failure if they result in increased exposure of concomitantly administered nephrotoxic drugs such as cyclosporin A, tacrolimus and sirolimus.^5^ The use of triazoles has also been associated with hepatotoxicity, hypokalaemia, cardiovascular events, neurotoxicity, phototoxicity, carcinomas/melanomas and a need for therapeutic drug level monitoring.^5,37,87,113^

### Echinocandins

The echinocandins are generally well tolerated, considered safer than the polyenes and triazoles and have no intrinsic nephrotoxicity. However, their approved indications are limited to invasive candidiasis and aspergillosis, with effectiveness in aspergillosis primarily as a component of combination antifungal therapy. They are also indicated for empirical use in persistently febrile patients with neutropenia.

## Conclusions

Invasive fungal diseases are deadly. Optimal antifungal treatment is required to increase the chance of survival in affected patients. Although LAmB is effective in managing invasive fungal infections, renal toxicity can occur, but it can be managed and is usually reversible. Progression to acute kidney failure with LAmB is rare.

It is important to understand the risk of nephrotoxicity associated with LAmB and the factors that increase this, and to be aware of strategies to mitigate this risk given the likelihood that LAmB will continue to play an important role in the management of invasive fungal disease.^118^ The number of patients at risk for invasive fungal infections and the rate of rare fungal infections are likely to increase, reinforcing the need for broad-spectrum antifungal agents. Difficulties in diagnosing invasive fungal infections are likely to persist, especially in mixed mould and mixed yeast infections, increasing the need for empirical and prophylactic treatment with broad-spectrum agents. Emerging patterns of antifungal resistance to azoles and echinocandins may challenge the effectiveness of these agents. Moreover, several new antifungal therapies show promising synergism with LAmB and are currently under evaluation in combination with LAmB.^119^ Together, optimal use of LAmB and emerging therapies has the potential to improve clinical outcomes and expand treatment options for patients with invasive fungal disease.

## Transparency declarations

JAM reports consulting fees, support for attending meetings or travel, participation and payment for service on advisory boards and honoraria from F2G; consulting fees from Cidara, Pfizer, Gilead Sciences, MSD, Takeda, Mundipharma, Basilea and Shionogi; honoraria from Cidara, Pfizer, Gilead Sciences, MSD, Takeda, Mundipharma, Basilea and Shionogi; support for attending meetings or travel from Cidara, Pfizer, Gilead Sciences, MSD, Takeda, Mundipharma, Basilea and Shionogi; participation and payment for service on advisory boards from Cidara and Basilea; and service as leadership or fiduciary role at ECIL (past chair). RB and TF report no conflicts of interest. DN reports research support and consulting fees from MSD, Pfizer, Takeda and Gilead Sciences. MH reports grants from Astellas, Scynexis, Gilead Sciences, IMMY, MSD, Pfizer, Euroimmun, Mundipharma, Melinta, Pulmocide and GSK; honoraria from Gilead Sciences, Onconova, Shionogi and Mundipharma; and research funding from Gilead Sciences, Astellas, Mundipharma, Euroimmun, MSD, GSK, Basilea, Pulmocide, Scynexis, AiCuris, Melinta, Partners, F2G, Shionogi, Stendahl and Pfizer.
